# Use of Chemical Indicators and Bioassays in Bottom Sediment Ecological Risk Assessment

**DOI:** 10.1007/s00244-018-0513-2

**Published:** 2018-02-27

**Authors:** Marek Tarnawski, Agnieszka Baran

**Affiliations:** 1 Department of Hydraulic Engineering and Geotechnics, University of Agriculture in Krakow, Al. Mickiewicza 24/28, 30-059 Kraków, Poland; 2 Department of Agricultural and Environmental Chemistry, University of Agriculture in Krakow, Al. Mickiewicza 21, 31-120 Kraków, Poland

## Abstract

This study is was designed to assess the ecological risk associated with chemical pollution caused by heavy metals and PAHs on the basis of their ecotoxicological properties in sediments collected from the Rzeszów dam reservoir (Poland). The sediment samples were collected from three sampling stations: S1—inlet, backwater station, S2—middle of reservoir, S3 outlet, near the dam. The sediments’ toxicity was evaluated using a battery of bioassays (Phytotoxkit, Phytotestkit, Ostracodtoxkit F, and Microtox). The highest content of metals (120.5 mg Zn; 22.65 mg Pb; 8.20 mg Cd ∙ kg^−1^ dw) and all PAHs (∑9361 μg ∙ kg^−1^ dw) in sediments was found at station S1. The lowest content of metals (86.72 mg Zn; 18.07 mg Cu; 17.20 mg Pb; 3.62 mg Cu; 28.78 mg Ni; 30.52 mg Cr ∙ kg^−1^ dw) and PAHs (∑4390 μg ∙ kg^−1^ dw) was found in the sediment from station S2. The ecological risk assessment of the six metals and eight PAHs revealed a high potential toxicity in sediments from stations S1 (PECq = 0.69) and S3 (PECq = 0.56) and a low potential toxicity in sediments from station S2 (PECq = 0.38). The studies also showed the actual toxicity of sediments for the test organisms. The sediment pore water was least toxic compared to the whole sediment: solid phases > whole sediment > pore water. The most sensitive organism for metals and PAHs in bottom sediments was *Lepidium sativum,* and in pore water—*Sorghum saccharatum.* The concentration of metals and PAHs in bottom sediments generally did not affect the toxicity for other organisms. Clay content and organic C content are likely to be important factors, which control heavy metal and PAH concentrations in the sediments. Data analysis by PCA found the same origin of metals as well as PAHs—mainly anthropogenic sources. The obtained information demonstrated the need to integrate ecotoxicological and chemical methods for an appropriate ecological risk assessment.

Bottom sediments are an important part of aquatic ecosystems (Förstner and Salomons 2010; Smal et al. 2015). They are a source of nutrients for microorganisms, plants and animal organisms; therefore, they constitute an important component of matter and the energy cycle in water reservoirs. Conversely, they are an important place and source of contamination for the aquatic food web (Cesar et al. 2006). Sediments may accumulate different mineral and organic contaminants at a concentration higher than the water column, thus producing harmful effects on the benthic organisms and for organisms, which feed on benthos. The pollution of the aquatic environment by heavy metals and PAHs has been known as one of the most challenging pollution issues due to the toxicity, persistence, and bioaccumulation (Buruaem et al. 2013; Fu et al. 2014; Li et al. 2015; Sukhdhane et al. 2015; Wang et al. 2016; Zhonghua et al. 2016). Many authors found that the analysis of the concentration of heavy metals and PAHs in sediments could be used to investigate anthropogenic impacts on the aquatic environment and assess the ecological risk (Höss et al. 2010; Buruaem et al. 2013; Ekere et al. 2017; Ezkwe and Utong 2017). Moreover, the chemical composition of bottom sediments is a good source of information about the human impact on the aquatic environment and an important indicator of the geochemical status in the river catchment (Roig et al. 2015; Urbaniak et al. 2015).

The assessment of the chemical status of sediments is important for identifying the type, concentration, and source of chemical substances and their metabolites, but it does not always show data about the effect of the pollution on organisms, and it does not provide any information on the synergic and antagonistic factors and their bioavailability (Wadhia and Thompson 2007; Mankiewicz-Boczek et al. 2008; Narracci et al. 2009; Tuikka et al. 2011, Witt et al. 2014, Buruaem et al. 2013; Baran and Tarnawski 2015). From the viewpoint of risk to the aquatic environment, it is important to determine to what extent pollution in sediments negatively impacts this environment, and if they may be considered as a stressor for organisms. Therefore, a challenge for the scientists dealing with aquatic risk assessment is to reveal the relations between the pollutants of sediments and the biological community response (Zotina et al. 2015; Kuzmanović et al. 2016). However, the biological status is not always in coherence with the chemical one (Roig et al. 2013). Few authors have explained this trend by the adaptation mechanisms of organisms under chronic exposure or regional specificities of the communities that cause high tolerance under extreme conditions (Wojtasik 2009; Roig et al. 2015; de Castro-Català et al. 2016). Therefore, the present ecotoxicological studies seem to be a good technique to integrate the biological response under the presence of different substances in nonadapted organisms. Bioassays are useful, cost-effective, and rapid tools, because their application enables a real assessment of the risk resulting from the presence of multiple chemical stressors in bottom sediments, their bioavailability, toxicity, and interaction (Latif and Licek 2004; Mankiewicz-Boczek et al. 2008; Goncalves et al. 2013; Besser et al. 2014; Baran and Tarnawski 2015). However, in ecotoxicological studies, an important factor is the selection of appropriate multiple tests—batteries of bioassays. It is crucial that the organisms belong to various taxonomic groups and represent various levels of the food chain (producers, consumers, and decomposers) (Blasco and Pico 2009; Höss et al. 2010; Tuikka et al. 2011; Baran and Tarnawski 2013; de Castro-Català et al. 2016).

The purpose of this study was (1) to assess the content of heavy metals and PAHs in sediments collected from the Rzeszów dam reservoir, (2) to evaluate the toxicity of sediments, sediment elutriates and pore water using four bioassays, and (3) to analyze a possible relationship between the observed toxicity and concentration of metals and PAHs. This study was designed not only to investigate the contents of heavy metals and PAHs in bottom sediments but also to assess their ecotoxicity and integrate the above-mentioned parameters in a complex assessment of risk associated with the occurrence of chemical substances in sediments.

## Materials and Methods

### Study Area and Sampling

The bottom sediment originated from the Rzeszów reservoir situated on the Wisłok River in the Podkarpackie Voivodeship, southeastern Poland. The Rzeszów reservoir was built as a result of the division of the Wisłok river valley (at km 63 + 760) by a barrage built in the early 1970s. Its total capacity is 1.8 m. m^3^, and its area is 68.2 ha. It has an elongated shape with a length of approximately 6.74 km and a width ranging from 40 to 600 m. Its main functions include: enabling a water intake for the Rzeszów city water supply system, flood control, and creating conditions for recreation for the inhabitants of the city and its surroundings (Michalec and Tarnawski 2008; Gruca-Rokosz and Tomaszak 2015; Baran et al. 2015). Together with tributaries and the reservoir bowl area, the middle course of the Wisłok River is under protection as part of the Natura 2000 program. Measurements taken after 14 years of the reservoir operation revealed that its capacity had diminished by approximately 66%, with a loss of 18.5 ha of impoundment area, constituting permanently or periodically overgrown islands and shallows (Koniarz et al. 2015). Currently, after 44 years of exploitation, as a result of excessive silting, the Rzeszów reservoir has lost a significant part of its original capacity. The result of the fill up is a partial loss of its two fundamental functions, i.e., a decline of the possibility of collecting water by surface intakes for the city of Rzeszów and industrial water and the possibility of using it for recreational and sport purposes. In 2010, a permit was issued allowing for the extraction of 1.5 m. m^3^ of bottom sediments (stretched over 3.6 km) by the silting up method. The works were performed with numerous time limits on account of fowl, mammals, fish, and amphibian close seasons. The obtained excavated material, depending on its granulometry, was used for construction works (gravel, sand) and on urban green areas after being mixed with peat (fine-grained sand, dust, silt). Based on the analysis, it was stated that the tested sediments can be preliminarily accepted for forming soil liners in municipal waste disposal sites (Koś and Zawisza 2012).

**Fig. 1:**
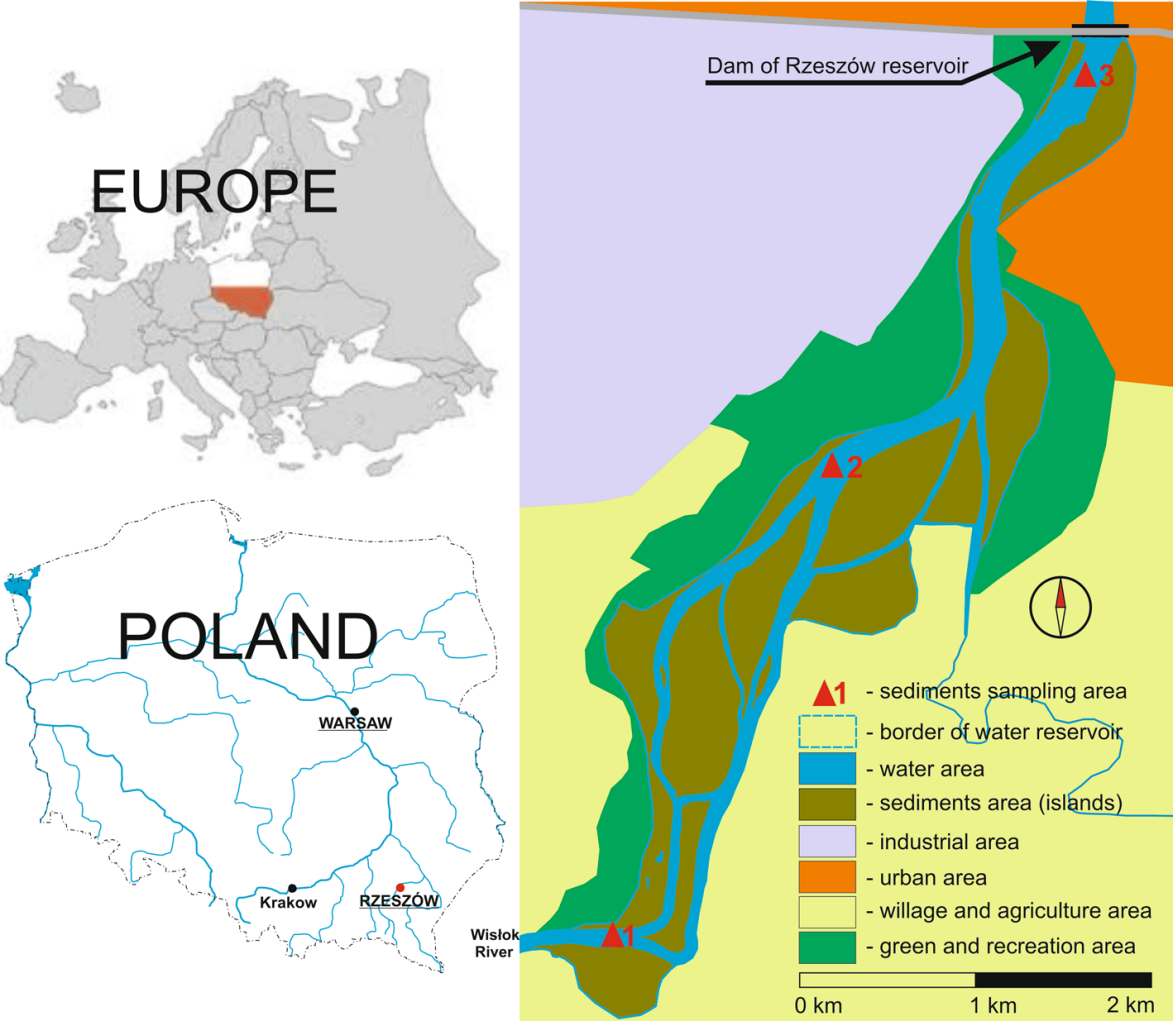
Location of the reservoir and three station samples

The sediment samples were collected from three sampling stations: S1 was located in the inlet (backwater station), S2 was situated in the middle of reservoir, and S3 was positioned outlet (near to dam) (Fig. 1). An Ekman sampler was used to collect sediment samples from the top layer (0–15 cm). Five or six samples were collected from each zone and mixed to average the properties of sediment samples representing individual zones (distance of 10–50 m^2^ area; from 2 to 4 dm^3^ volume of each subsample) and bulked together to form one composite sample. Pore water was obtained by the centrifugation (5000 rpm, 15 min.) of sediments, and placed in 100 cm^3^ polypropylene conical test tubes. The pore water samples and sediment samples were refrigerated until they were analyzed (Baran and Tarnawski 2013).

### Chemical Analysis

Basic parameters, such as particle size distribution, pH, conductivity, organic C, and total nitrogen, were analyzed in the sediment samples (Baran and Tarnawski 2015). The total heavy metal concentration in the sediment and pore water was analyzed using the ICP-OES (Inductively Coupled Plasma Optical Emission Spectroscopy) method on Optima 7300 DV (Perkin-Elmer), as described previously (Baran and Tarnawski 2013, 2015). The analytical results of the quality control samples showed a good agreement with the certified value reference material (CRM 16-050), recoveries ranging from 93.6 (Cd) to 105.4% (Ni). The concentrations of the eight PAHs [naphthalene (NAP), phenanthrene (PHE), anthracene (ANT), fluoranthene (FLT), pyrene (PYR), benzo(a)anthracene BAA, chrysene CHR, and benzo(a)pyrene (BAP)] were determined by gas chromatography–mass spectrometry on a Varian GC/MS/MS 4000 equipped with an ion trap. A detailed description of the analytical procedure was previously described by Baran et al. (2017). The recoveries ranged from 80% (CHR) to 98.5% (NAP). In addition, the limit of detection (LOD) was 2 µg · kg^−1^ dw, and the limit of quantification (LOQ) was 6 µg · kg^−1^ dw (Baran et al. 2017).

### Ecotoxicity Tests

Four bioassays were used to assess the bottom sediments and the pore water toxicity: Phytotoxkit (sediment), Phytotestkit (pore water), Ostracodtoxkit F (sediment), and Microtox (sediment elutriate, pore water). Two parameters were measured in the Phytotoxkit and Phytotestkit bioassay: the inhibition of seed germination (IG) as well as root length inhibition (IR) of *Sinapis alba*, *Lepidium sativum* and *Sorghum saccharatum* (Phytotoxkit 2004). In the Ostarcodtoxkit test, the toxicity of samples was also assessed using two endpoints: the mortality and the growth inhibition of crustacean *Heterocypris incongruens* (Ostracodtoxkit 2001). A decrease in the luminescence of bacteria *Vibrio fischeri* (Microtox^®^) was used to assess the toxicity of the sediment and pore water samples. The measurement of the change in luminescence was performed with an 81.9% Screening Test using the Microtox M500 Analyzer (MicrobicsCorporation 1992). The bioassays were carried out in 3 (Phytotoxkit, Phytotestkit, Microtox) and 6 (Ostracodtoxkit) repetitions. The tests were performed according to standard procedures (Phytotoxkit 2004, Ostracodtoxkit 2001, MicrobicsCorporation 1992). The test methods were described in our previous studies (Baran and Tarnawski 2013, 2015). The toxicity results were presented as a Percent effect (PE %) and classified into one of five classes (Matejczyk et al. 2011).

### Potential of Ecological Risk Assessment

The concentrations of heavy metals and PAHs in bottom sediments were compared with the consensus-based sediment quality guideline values referred to as TEC (Threshold Effect Concentration) and PEC (Probable Effect Concentration), (Macdonald et al. 2000). The hazard quotient (HQ) was calculated based on the individual metal and PAH concentration as well as the concentration to its corresponding PEC (Khairy et al. 2009):

$${\text{HQ}} = \frac{C}{\text{PEC}}$$

*C* represents the measured values of heavy metals or PAHs in the sediments, and PEC is the Probable Effect Concentration (Macdonald et al. 2000).

All HQ in the pore water have been calculated by using the freshwater US EPA screening benchmarks for aquatic ecosystem protection (Roig et al. 2015). When HQ > 1, frequent adverse ecological effects are excepted (Khairy et al. 2009). In addition, the mean PEC quotient (PECq) for seven metals and eight PAHs was calculated as:

$${\text{PECq}} = \frac{{\sum {\frac{C}{\text{PEC}}} }}{n}$$

*C* represents the measured values of heavy metals or PAHs in the sediments, PEC is the Probable Effect Concentration (Macdonald et al. 2000), and n is the number of tested chemical compounds.

The mean PECq was the average ratio of each metal and PAH concentration to its corresponding PEC. The sediment samples were predicted to be nontoxic, indicating a low potential toxicity to the benthic fauna when the mean PECq was < 0.5. If the mean PECq was > 0.5, the sediments were toxic, pointing out a high potential risk to the benthic fauna (Perrodin et al. 2006).

### Statistical Analysis

Statistical analysis was performed and involved determination of mean, standard deviation. The relationships between the analyzed parameters (chemical and ecotoxicological) in sediments were assessed using Pearson correlation coefficients and PCA. A Microsoft Excel 2007 spreadsheet and the Statistica 12.5 package were used for the analysis and presentation of the obtained results.

## Results and Discussion

### Physicochemical Properties of Bottom Sediments

The sediments showed a neutral as well as an alkaline reaction, and a pH was ranging from 7.06 (S1) to 7.18 (S2). The electrolytic conductivity ranged from 0.14 (S3) to 0.34 mS ∙ cm^−1^ (S2). The content of organic C in the bottom sediments was low and within the range from 1.98 (S2) to 2.89% (S1). Carbon to nitrogen ratios (C/N) were between 9.97 (S1) and 11.35 (S2). Generally, clay fraction [48% (S2)–57% (S1)] were dominant in the bottom sediments collected from the Rzeszów reservoir. Based on the grain size fraction, the bottom material contains from 7% (S2) to 9% (S3) sand and from 35% (S1, S3) to 45% (S2) silt.

The concentration of the total heavy metal content in the sediments was between 86.72 and 120.5 mg Zn; between 18.07 and 24.20 mg Cu; between 30.52 and 40.07 mg Cr; between 17.20 and 22.65 mg Pb; between 2.92 and 8.20 mg Cd; between 28.78 and 38.10 mg Ni ∙ kg^−1^ dw (Table 1). Station S1 showed the highest Zn, Pb and Cd concentrations in the bottom sediments. The highest Cu, Cr and Ni concentrations were found in the bottom sediment from station S3. The lowest concentration of Zn, Cu, Pb, Ni, and Cr was found in the sediment from station S2, and Cd—from S3. Sediment contamination with heavy metals was assessed based on Bojakowska’s geochemical quality classes of bottom sediments (Bojakowska 2001). The above classification distinguishes four classes: I—noncontaminated sediments, II—moderately contaminated sediments, III—highly contaminated sediments, and IV—very highly contaminated sediments. According to this criterion, the sediment samples were classified as class II (moderately contaminated sediments) due to an elevated concentration of Cu (S2, S3), Cd (S1) and Ni (all stations). A relatively high concentration of Ni and Cd is a good indicator of a recent anthropogenic pollution (Ekere et al. 2017). Nickel is commonly used in industry and it can be found at high concentration in freshwater areas surrounding developed urban areas (de Castro-Català et al. 2016).

**Table 1:** Total heavy metal concentration in bottom sediment

Station	Zn	Cu	Pb	Cd	Ni	Cr
*Sediment (mg ∙ kg* ^*−1*^ *dw)*						
S1	120.5 ± 2.72	20.15 ± 0.71	22.65 ± 0.81	8.20 ± 0.42	33.53 ± 0.49	35.90 ± 3.32
S2	86.72 ± 1.77	18.07 ± 0.14	17.20 ± 0.28	3.67 ± 0.32	28.78 ± 0.53	30.52 ± 1.84
S3	113.3 ± 5.23	24.20 ± 1.63	20.18 ± 1.24	2.92 ± 0.18	38.10 ± 0.35	40.07 ± 2.16
TEC/PEC^a^	121/459	31.6/149	35.8/128	0.99/4.98	22.7/48.6	43.3/111
*Pore water (µg ∙ dm* ^*−3*^ *)*						
S1	122.6 ± 6.72	8.66 ± 0.57	0.60 ± 0.02	nd	9.99 ± 0.69	15.70 ± 1.0
S2	128.8 ± 13.0	9.28 ± 0.45	2.34 ± 0.61	nd	12.90 ± 0.99	18.25 ± 0.3
S3	81.95 ± 7.70	12.86 ± 0.67	1.64 ± 0.30	nd	9.79 ± 0.63	16.64 ± 1.14
US EPA^a^	120	9	2.5	0.25	52	85

*nd* Not detected

^a^Macdonald et al. (2000) and Roig et al. (2015)

The concentration of the metals in pore water was low as compared to the total concentration in the sediment. The metal concentration in the pore water ranged (µg ∙ dm^−3^) from 81.95 to 128.8 µg Zn; from 8.66 to 12.86 µg Cu, from 15.70 to 18.25 µg Cr; from 0.60 to 2.34 µg Pb; from 9.79 to 12.90 µg Ni. However, cadmium concentration was below the detection limit in the pore water (Table 1). In the study by Roig et al. (2015), a lower concentration of Zn, Pb was found, along with a higher concentration of Cr, Cu, Ni, and Cd in pore water compared to our study. The concentrations of metals in pore water ranged from < 0.02 to 0.06 μg Cd; from 34.8 to 223 μg Cr; from 2.82 to 94.6 μg Cu; from 0.26 to 14.96 μg Ni; from < 0.02 to 0.11 μg Pb, and from < 0.2 to 10.7 μg Zn ∙ dm^−3^ (Roig et al. 2015). The concentration of Zn (S1, S2), Cu (S2, S3) in the pore water slightly exceeded the standards for surface water (Table 1).

The concentrations of each PAH and the total PAH in the bottom sediments are given in Table 2. The PAH concentrations in the sediments taken from the three sampling stations varied in a different extent depending on the type of substance: 40–50 μg NAP; 350–530 μg PHE; 40–90 μg ANT; 1100–2260 μg FLT; 970–1840 μg PYR; 410–810 μg BAA; 730–1380 μg CHR; and 1180–2400 μg BAP ∙ kg^−1^ dw. The∑PAHs concentration in the surface sediments ranged from 4820 to 9350 µg ∙ kg^−1^ dw. The highest concentration of PAHs was found in sediments taken from the Rzeszów reservoir from station S1 (inlet, backwater station), and the lowest one from station S2. In the literature, we found four categories of ∑PAH pollution levels in bottom sediments: low (0–100 µg · kg^−1^ dw), moderate (100–1000 µg · kg^−1^ dw), high (1000–5000 µg · kg^−1^ dw), and very high (> 5000 µg · kg^−1^ dw) (Baumard et al. 1999). Sediments from the Rzeszów reservoir had high concentrations of PAHs (III class). The reservoir is under strong human pressure associated with industry, long-range transport of PAHs and local agriculture, which causes severe erosion of the land (Koniarz et al. 2015; Bartoszek et al. 2015; Baran et al. 2017). Bartoszek et al. (2015) showed that the greatest impact on the quality of bottom sediments is connected with the presence of BAP. The concentrations of BAP in the obtained samples also were high, in the range of 1180–2400 μg ∙ kg^−1^ dw. Moreover, BAP and FLT were the major compounds constituting, on average, approximately 26 and 23% of total PAHs, followed by PYR and CHR—19 and 14% of total PAHs, respectively.

**Table 2:** PAHs concentration in bottom sediment

Station	NAP	PHE	ANT	FLT	PYR	BAA	CHR	BAP	∑PAH	LWM	HWM
	µg ∙ kg^−1^ dw										
S1	51 ± 10	530 ± 25	90 ± 14	2260 ± 100	1840 ± 44	810 ± 38	1380 ± 108	2400 ± 109	9361	671	8690
S2	40 ± 2	340 ± 26	40 ± 11	1100 ± 58	970 ± 25	410 ± 39	730 ± 52	1180 ± 85	4810	420	4390
S3	50 ± 7	520 ± 46	87 ± 13	1770 ± 85	1440 ± 52	750 ± 19	1080 ± 110	2350 ± 56	8047	657	7390
TEC^a^	176	204	57.2	423	195	108	166	150	–	–	–
PEC^a^	561	1170	845	2230	1520	1050	1290	1450	–	–	–

^a^Macdonald et al. (2000)

### Assessment of Ecological Risk

The sediments were predicted to be non-toxic if the concentrations of heavy metals and PAHs were lower than the TECs (Threshold Effect Concentrations) (Tables 1, 2). However, if the concentrations of metals and PAHs were higher than the PECs (Probable Effect Concentration), the sediments were predicted to be toxic (Tables 1, 2). Sediments with contaminant concentrations between the TEC and PEC were predicted to be neither toxic nor nontoxic (Macdonald et al. 2000). In the case of nickel and cadmium, TEC values were exceeded, and PEC values for cadmium were exceeded in all the stations. For other metals, their concentrations in the bottom sediments were lower than TEC values (Table 1). For individual PAHs concentration in sediments, such as PYR, FLT, CHR (station S1), and BAP (stations S1, S3), a higher possibility of the occurrence of an adverse ecological effect was indicated. The concentrations of PHE, BAA (all stations), FLT, CHR, PYR (stations S2, S3), ANT (stations S1, S3), BAP (station S2) in the sediments were between the TEC and PEC values, indicating a lower possibility of the occurrence of an adverse ecological effect (Table 2). Moreover, we found that NAP (all stations) and ANT (station S2) are not contaminants of any major concern (Table 4). The value of the hazard quotients (HQ) in the sediment > 1 was observed only for Cd, FLT, PYR, CHR (station S1), and BAP (stations S1, S2). The mean PECq of heavy metals and PAHs ranged from 0.38 to 0.69 (Table 3). The highest value of PECq was found for sediments from station S1 (inlet) and the lowest value for sediments from station S2 (middle). Generally, the risk assessment revealed that the total content of metals and PAHs was likely to cause a high potential toxicity to biological communities (PECq > 0.5) in sediments from stations S1 and S3. A low potential toxicity to the benthic fauna (PECq < 0.5) was shown for sediments from station S2. Other authors have divided PEC quotients into four categories: non-adverse effect (PECq < 0.1), slightly adverse effect (0.1 < PECq < 0.5), moderate effect (0.5 < PECq < 1.0), and heavy effect (PECq > 1.0) (Ingersoll et al. 2001; Tavakoly Sany et al. 2014). Based on these ranges, a slightly adverse effect on the benthic fauna was shown for sediments from station S2, whereas a moderate effect was found for sediments from stations S1 and S3 (Table 3). Considering the value of HQ for metals in pore water, Zn (S2) and Cu (S2, S3) were the elements that contributed the most to the risk. A low risk connected with metals (PECq < 0.5) was shown for pore water from station 1, and a high risk (PECq > 0.5)—from stations S2, S3.

**Table 3:** Hazard quotients calculated for chemical substance in the sediment and pore water

Station	Zn	Cu	Pb	Cd	Ni	Cr	NAP	PHE	ANT	FLT	PYR	BAA	CHR	BAP	PECq^a^
*Sediment*															
S1	0.26	0.14	0.18	1.65	0.69	0.32	0.09	0.45	0.11	1.01	1.21	0.77	1.07	1.66	0.69
S2	0.19	0.12	0.13	0.74	0.59	0.27	0.07	0.29	0.05	0.49	0.64	0.39	0.57	0.81	0.38
S3	0.25	0.16	0.16	0.59	0.78	0.36	0.09	0.44	0.10	0.79	0.95	0.71	0.84	1.62	0.56
*Pore water*															
S1	0.95	0.96	0.24	0.00	0.19	0.18	–	–	–	–	–	–	–	–	0.42
S2	1.07	1.03	0.94	0.00	0.25	0.21	–	–	–	–	–	–	–	–	0.58
S3	0.68	1.43	0.66	0.00	0.19	0.20	–	–	–	–	–	–	–	–	0.53

^a^Mean PEC quotient

### Sediment and Pore Water Toxicity

Plant germination inhibition ranged from 0 to 40% (solid phases), from 0 to 33% (pore water), and from 0 to 30% (whole sediment). The inhibition of root growth was between 8 and 76% (solid phases), between − 11 and 32% (pore water), and between − 5 and 62% (whole sediment) (Table 4). *H. incongruens* mortality varied between 0 and 15% and the growth inhibition ranged from − 6 to 37% (Table 4). *V. fischeri* luminescence inhibition was from 9 to 18% (solid phases), from 8 to 9% (whole sediment), and from − 21 to 30% (pore water) (Table 4). All bottom sediments were classified as toxicity class III (S1, S3) and II (S2) (Table 5). The sediments’ solid phases exhibited class III (S 1) and II (S2, S3). The least toxicity was found for the pore water, which was classified as toxicity class I (S1, S2) and II (S3). Sediments from station S2 showed the lowest toxicity to the test organisms (Table 4), and sediments from station S1 had the highest toxicity (Table 4). Generally, more toxicity was found in sediment bioassays than in pore water bioassays. The mean toxicity of the samples can be placed in the following order: solid phases > whole sediment > pore water. Our results are in agreement with the studies of Baran and Tarnawski (2013, 2015) as well as de Castro-Català et al. (2016). However, some authors have indicated that the pore water test is more likely to detect toxicity than the solid-phase tests (Winger et al. 2000; Roig et al. 2015). The study of Buruaem et al. (2013) showed that pore water is more toxic than the whole sediment. The authors suggest that the test organisms in pore water are exposed to dissolved contaminants, which are easily absorbed by diffusion through their body surface. Pore water is a space filled with water between sediment grains, which constantly remains in contact with sediments; therefore, it may exchange pollutants between them. This makes pore water a valuable tool for the assessment of the potential bioavailability of chemical substances or their toxicity (Baran and Tarnawski 2015; Roig et al. 2015). Moreover, pore water is more concentrated than surface waters, but it is important to emphasize that benthic organisms are highly exposed to it (de Castro-Català et al. 2016). Generally, plants showed the highest sensitivity compared to other organisms. In the study, the lowest sensitivity was observed in *H. incongruens* (solid phases, whole sediment) and *S. alba* (pore water) (Table 4). The greatest number of toxic responses in the whole sediment was found for *L. sativum* (root inhibition); in the solid phases—for *L. sativum* and *S. saccharatum* (root inhibition); and in pore water—for *S. saccharatum* (germination inhibition and root inhibition). We found that *L. sativum* was the most sensitive plant species (bottom sediment) along with *S. saccharatum* (pore water). This result is in agreement with our early study focusing on the toxicity of bottom sediments from the Rybnik reservoir (Baran and Tarnawski 2015). However, in the studies of Czerniawska-Kusza et al. (2006) and Baran and Tarnawski (2013), it was reported that *S. saccharatum* is the most sensitive plant compared with *L. sativum* and S*. alba.* Among the test plants, *S. alba* was the least sensitive to the concentration of chemical substances in the bottom sediments. Similar results were obtained in the studies on the toxicity of sediments from the Zesławice reservoir (Baran and Tarnawski 2013). Mankiewicz-Boczek et al. (2008) presented the highest number of toxic responses in the Phytotoxkit test, followed by the Microtox^®^ and the Ostracodtoxkit. F. Roig et al. (2011, 2015) and Kudłak et al. (2011) found that algae tests seem to be more sensitive than other organisms in pore water and bottom sediment samples. We think that the reason for the high sensitivity of plants compared to *V. fischeri* and *H. incongruens* is related to their growth conditions. The growth of a plant can be limited both by toxicants and by nutrient deficiencies in the bottom sediment. Zotina et al. (2015) found the limitations of the *Elodea* shoot and root growth under a nutrient deficiency. However, Lahr et al. (2003) indicated that crustaceans *T. platyurus* and *D. magna* were the most sensitive organisms in a battery of bioassays with pore water. In the study of Roig et al. (2015), among different bioassays performed in the whole bottom sediment samples, *Chironomus riparius* was the most sensitive organism.

**Table 4:** Toxicity of bottom sediment

Station	Phytotoxkit						Ostracodtoxkit F		Microtox
	Germination inhibition			Roots growth inhibition			Mortality	Growth inhibition	Luminescence
	Ls	Sa	Ss	Ls	Sa	Ss	*H. incongruens*		*V. fischeri*
Percent effect PE %									
Whole bottom sediments									
S1	0 ± 0	0 ± 0	30 ± 0	60 ± 2.8	9 ± 1.6	15 ± 2.5	0 ± 0	− 6 ± 3	8 ± 1.4
S2	20 ± 0	0 ± 0	15 ± 0	40 ± 0.9	22 ± 2.0	5 ± 1.0	0 ± 0	− 4 ± 0.5	9 ± 1.2
S3	30 ± 0	0 ± 0	10 ± 0	54 ± 1.5	62 ± 3.7	− 5 ± 0.9	5 ± 0.5	− 5 ± 0.8	8 ± 0.6
Solid phases of sediments									
S1	18 ± 3	20 ± 0	40 ± 0	76 ± 0.6	35 ± 9.0	64 ± 0.4	5 ± 0.5	19 ± 7	9 ± 3
S2	14 ± 0	0 ± 0	30 ± 0	48 ± 0.8	8 ± 1.0	47 ± 0.3	15 ± 2	37 ± 3	18 ± 0.5
S3	4 ± 0	10 ± 0	0 ± 0	46 ± 2.9	24 ± 8.0	44 ± 1.3	15 ± 2	21 ± 8	9 ± 3
Pore water									
S1	8 ± 0	0 ± 0	33 ± 0	− 11 ± 1.2	1 ± 0.2	12 ± 0.4	–	–	− 21 ± 6
S2	9 ± 0	0 ± 0	0 ± 0	20 ± 2.6	8 ± 1.4	6 ± 1.5	–	–	− 8 ± 4
S3	0 ± 0	0 ± 0	22 ± 0	19 ± 2.7	− 3 ± 0.7	32 ± 1.3	–	–	30 ± 5

*Ls L. sativum, Sa S. alba, Ss S. saccharatum*

**Table 5:** Hazard classification

Samples	Station	Class^a^	Toxicity
Whole bottom sediments	S1	III	Acute toxicity
	S2	II	Slight acute toxicity
	S3	III	Acute toxicity
Solid phases of sediments	S1	III	Acute toxicity
	S2	II	Slight acute toxicity
	S3	II	Slight acute toxicity
Pore water	S1	I	No acute toxicity
	S2	I	No acute toxicity
	S3	II	Slight acute toxicity

^a^Class: I no acute toxicity PE < 20%; II slight acute toxicity 20% ≤ PE < 50%; III acute toxicity 50% ≤ PE < 75%; IV high acute toxicity 75% ≤ PE < 100%; very high acute toxicity PE ≥ 100%

### Correlation Coefficient and PCA Analysis

**Fig. 2:**
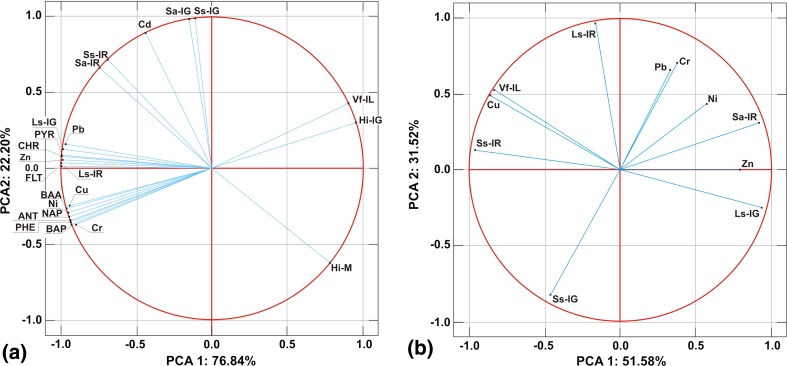
Results of PCA relationships between chemical and ecotoxicological factors in bottom sediments (**a**) and pore water (**b**). *IG Ls* inhibition of germination of *L. sativum*; *IR Ls* inhibition of root growth of *L. sativum*; *IG Sa* inhibition of germination of *S. alba*; *IR Sa* inhibition of root growth of *S. alba*; *IG Ss* inhibition of germination of *S. saccharatum*; *IR Ss* inhibition of root growth of *S. saccharatum*; *M Hi* mortality of *H. incongruens*; *IG Hi* inhibition of growth of *H. incongruens*; *IL Vf* inhibition of luminescence of *V. fischeri*

The concentrations of heavy metals (Cr, Ni, Zn, Cu, Pb) and all PAHs were significantly positively correlated with clay content and significantly negatively correlated with silt (Table 6). We think that the high positive correlation between metals, PAHs, and clay content is due to the degree of the silting of reservoirs. The intensive silting process contributes to the inflow of fine (mineral or organic) fractions of both natural and anthropogenic origin. Fine fractions have high sorption capacities for metals and PAHs (Dahle et al. 2003). Moreover, the clay content is the dominant fraction in the bottom sediments of the Rzeszów reservoir. We also found a significantly positive correlation between the concentrations of Cr, NAP, PHE, ANT, BAP, and sand and between the concentrations of Ni, Cu, all PAHs, and organic C. The concentrations of metals and PAHs did not correlate with the values of the sediment pH. Many authors have found that the positive correlations of metals and PAHs with the organic carbon content in the sediments might be attributed to anthropogenic impacts (Shaheen and Rinklebe 2014; Baran et al. 2016). Organic carbon is an important parameter governing the distribution and adsorption of PAHs in bottom sediments (Dahle et al. 2003; Wang et al. 2013). A strong relationship between PAH concentrations and organic matter content or clay fraction in bottom sediments is often observed (Oros and Rosa 2004; Khairy et al. 2009; Lubecki and Kowalewska 2010; Baran et al. 2017). Moreover, relationships among the individual metals were significantly, positively correlated with one another and with the PAHs (except Cd) (Fig. 2a). A similar relationship was found for PAHs (Fig. 2a). Strong positive correlations between individual pairs of heavy metals and PAHs indicate that they have a similar source and move together to the reservoir (Rinklebe and Shaheen 2014; Shaheen and Rinklebe 2014; Baran et al. 2016; Ekere et al. 2017). There are two main sources of PAH contaminations: petrogenic and pyrolytic. Generally, petrogenic PAHs are characterized by the predominance of low molecular weight PAHs (2- and 3-ring PAHs), while pyrogenic PAHs are characterized by a higher molecular weight PAHs (above 4-ring PAHs) (Kafilzadeh 2015). Moreover, the ratio between PAHs was used to identify possible PAHs sources in the samples. The ratio between LMW/HMW < 1 (low molecular weight PAHs/high molecular weight PAHs) implies PAHs of a pyrolytic origin (Tavakoly Sany et al. 2014; dos Santos et al. 2017). Pyrolytic sources comprise an incomplete combustion of organic compounds, such as fossil fuels, whereas the petrogenic PAHs are formed by petroleum products. We found that 4-ring PAHs were prevalent in the sediments form the Rzeszów reservoir (66% of total PAHs). The HMW PAHs contributed between 91 and 93% of the overall concentration of PAHs in the sediments, which implies the domination of pyrogenic sources. BAP often is used as an indicator for PAHs originating from combustion (Ekere et al. 2017). In the study, a high correlation between BAP and other PAHs was found, which indicates that combustion is the primary sources of the PAHs (Fig. 2a).

**Table 6:** Relationships between toxicity and chemical properties of sediments

	Sand	Silt	Clay	pH	C-org	IG Ls	IR Ls	IG Sa	IR Sa	IG Ss	IR Ss	M Hi	IG Hi		IL Vf
Cr	0.81*	− 0.96*	0.96*	− 0.64	0.78	0.85*	0.89*	− 0.2	0.46	− 0.23	0.39	− 0.51		− 0.96*	− 0.96*
Ni	0.79	− 0.99*	0.99*	− 0.44	0.81*	0.91*	0.95*	− 0.13	0.54	− 0.17	0.47	− 0.59		− 1.00*	− 0.99*
Zn	0.55	− 0.89*	0.93*	− 0.28	0.77	0.99*	0.99*	0.21	0.79	0.18	0.74	− 0.83*		− 0.93*	− 0.88*
Pb	0.45	− 0.82*	0.87*	− 0.36	0.74	0.97*	0.96*	0.31	0.64	0.28	0.79	− 0.87*		− 0.87*	− 0.80*
Cu	0.76	− 0.96*	0.97*	− 0.51	0.81*	0.91*	0.94*	− 0.09	0.56	− 0.13	0.5	− 0.61		− 0.97*	− 0.96*
Cd	− 0.42	− 0.07	0.17	0.36	0.16	0.55	0.46	0.93*	0.90*	0.92*	0.93*	− 0.88*		− 0.17	− 0.04
NAP	0.82*	− 0.98*	0.98*	− 0.48	0.84*	0.90*	0.94*	− 0.16	0.52	− 0.2	0.45	− 0.57		− 0.98*	− 0.99
PHE	0.84*	− 0.96*	0.97*	− 0.49	0.83*	0.89*	0.93*	− 0.19	0.49	− 0.23	0.42	− 0.54		− 0.97*	− 0.97*
ANT	0.84*	− 0.97*	0.98*	− 0.49	0.83*	0.89*	0.93*	− 0.19	0.49	− 0.23	0.42	− 0.55		− 0.99*	− 0.98*
FLT	0.58	− 0.91*	0.95*	− 0.35	0.82*	1.00*	1,00*	0.19	0.78	0.15	0.73	− 0.82*		− 0.95*	− 0.89*
PYR	0.54	− 0.89*	0.93*	− 0.33	0.81*	1.00*	1,00*	0.23	0.81	0.19	0.76	− 0.84*		− 0.93*	− 0.87*
BAA	0.79	− 0.99*	0.98*	− 0.47	0.84*	0.93*	0.96*	− 0.1	0.57	− 0.14	0.5	− 0.62		− 0.98*	− 0.99*
CHR	0.54	− 0.89*	0.93*	− 0.33	0.81*	1.00*	1,00*	0.23	0.61	0.2	0.76	− 0.84*		− 0.93*	− 0.87*
BAP	0.85*	− 0.97*	0.98*	− 0.50	0.83*	0.88*	0.93*	− 0.2	0.48	− 0.24	0.41	− 0.53		− 0.98*	− 0.97*
PECQ	–	–	–	–	–	0.98*	0.98*	0.19	0.78	0.15	0.73	− 0.82*		− 0.95*	− 0.90*

*IG Ls* inhibition of germination of *L. sativum*, *IR Ls* inhibition of root growth of *L. sativum*, *IG Sa* inhibition of germination of *S. alba*, *IR Sa* inhibition of root growth of *S. alba*, *IG Ss* inhibition of germination of *S. saccharatum*, *IR Ss* inhibition of root growth of *S. saccharatum*, *M Hi* mortality of *H. incongruens*, *IG Hi* inhibition of growth *of H. incongruens*, *IL Vf* inhibition of luminescence of *V. fischeri*

*Statistical significant at *p* < 0.05

In the pore water, we found a significantly positive correlation only between Ni and Zn and Pb (*r* = 0.84, *r* = 0.71, *p* < 0.05, respectively) and a significantly negative correlation between Cu and Zn (*r* = 0.78, *p* < 0.05; Fig. 2b).

Some correlations were performed to relate the concentrations of metals and PAHs in the sediments and the results of toxicity to the test organisms (Table 6; Fig. 2). For instance, the inhibition of germination and root growth of *L. sativum* in the sediments correlated significantly, positively with Cr, Ni, Zn, Pb, Cu, and PAHs; *S. alba* and *S. saccharatum* inhibition of germination and root growth correlated significantly, positively with Cd (Table 6). Most relations of *H. incongruens* and *V. fischeri* were negative. It indicates that the concentration of the metals and PAHs in bottom sediments did not affect their toxicity to the test organisms (Table 6). The above data are in indirect concordance with the HQ value. According to the HQ, substances that contributed more to the overall toxicity of the sediments were Cd followed by Ni and BAP, followed by PYR, CHR, and FLT (Table 3). Considering the correlations between mean PECqs and the ecotoxicity effects of bottom sediments, a significantly positive correlation with *L. sativum* and a significantly negative correlation with *H. incongruens* and *V. fischeri* (Table 6) were found. Our other study revealed a significant and positive correlation between mean PECqs, and the luminescence inhibition of *V. fischeri* as well as growth inhibition of *H. incongruens* (Baran et al. 2016). A significant correlation was found between luminescence inhibition in *V. fischeri* and growth inhibition in *H. incongruens* (*r* = 0.99, *p* < 0.05) as well as between germination and root growth inhibitions of *S. alba* and *S. saccchartum* (*r* = 0.99, *p* < 0.05). Therefore, it would seem that the correlation analysis showed a similar sensitivity to toxicants in the sediments. Generally, relations between the reaction of test plants and the reaction of *H. incongruens* or *V. fischeri* were negative (Fig. 2b). The study did not find many relationships with pore water. In the pore water, a significantly positive correlation was observed only between Cu and root inhibition of *S. saccharatum* and luminescence inhibition of *V. fischeri* (*r* = 0.89, *r* = 0.99, *p* < 0.05, respectively) as well as a significantly negative correlation between Cu, Cr and germination inhibition of *L. sativum* and *S. saccharatum* (*r* = − 0.93, *r* = − 0.85, *p* < 0.05, respectively). This is because Cu in pore water seems to be the most critical element. We also found that Cu was the most critical element in pore water regarding HQ (Table 3). This observation is in concordance with Khangarot and Ray (1989) and Roig et al. (2013). In the pore water, we also found a significant correlation between root growth inhibition of *S. alba* and germination inhibition of *L. sativum* (positive) as well as root growth inhibition of *S. saccharatum* (negative) (Fig. 2b). Germination inhibition of *L. sativum* was significantly, negatively correlated with root growth inhibition of *S. saccharatum* and *V. fischerii*. Moreover, root growth inhibition of *S. saccharatum* was significantly, positively correlated with the reaction of *V. fischerii* (Fig. 2b).

There have not been many reactions with pore water and bottom sediment bioassays, but a total consent between the solid phase and the pore water toxicity test is not necessarily desirable (Roig et al. 2015). Doe et al. (2003) found that different levels of agreement between the solid phase and the pore water bioassay are to be expected when toxicity is due to a moderate concentration of contamination, as in our present study. Moreover, not many significant correlations between the total metal concentrations in bottom sediments and the pore water metal concentration have been observed. We found a significantly negative correlation between the concentration of Cr in pore water and Zn, Ni, Pb, and Cu in bottom sediments (*r* = from − 0.82 to − 0.89, *p* < 0.05, respectively). Total metal concentration in sediments is generally unrelated to the most bioavailable fraction of metals in pore water (Baran and Tarnawski 2015; Roig et al. 2015). The main factors affecting the mobility and bioavailability of heavy metals from bottom sediments include reaction, organic matter content and grain size distribution (Prokop et al. 2003; Peakall and Burger 2003; Du Laing et al. 2009; Czerniawska-Kusza and Kusza 2011; de Castro-Català et al. 2016). Bottom sediments from the Rzeszów reservoir were classified as a group of clay deposits with alkaline reaction. Therefore, these properties might have influenced the poor solubility and mobility of the heavy metals from the solid phase of sediments to pore water. Our previous study found a low leachability of metals from the Rzeszów bottom sediments (Baran et al. 2015).

The analysis of the main components partially confirmed the previous observations. There were four distinct groups of compounds in the bottom sediments (Fig. 2a). The first and most numerous groups of factors or characteristics of the bottom sediment include: Cu, Ni, Cr, BAA, PHE, BAP, ANT, and NAP. The second group consists of Zn, Pb, PYR, CHR, FLT, Ls-IR, and Ls-IG. The third group consists of Cd, Sa-IR, Sa-IG, SS-IR, and Ss-IG. The last and least numerous group includes Vf-L, Hi-IG, and Hi-M (Fig. 2a). The first group and the second group, although they form separate groupings on the chart, also remain positively correlated with each other, but it is not as strong of a correlation as in the case of intergroup parts. The third group does not show any significant correlation, positive or negative, with the two previous groups. The fourth group is, in general, significantly, negatively correlated with the two previous groups. It was established that the first, second, and fourth groups are the main components of the first factor (PCA1). This factor accounted for 76.84% of the variance (Fig. 2a). This factor correlated with the concentrations of metals (without Cd), PAHs, and toxicity to *L. sativum, V. fischerii,* and *H. incongruens*. The third group (Cd, toxicity to *S. saccharatum* and *S. alba*) is a component of the second factor (Fig. 2a). In the pore water, the PCA1 accounted for 51.58% of the variance, and correlated with the concentrations of Cu, Cr, Zn, toxicity to *S. alba* (Sa-IR), *L. sativum* (Ls-IG), *S. saccharatum* (Ss-IR), and *V. fischeri* (Fig. 2b). The second factor (PCA2) accounted for 31.52% of the variances and the combined concentration of Pb, Cr and toxicity to *L. sativum* (LS-IR) and *S. saccharatum* (Ss-IG) (Fig. 2b). The first two primary factors accounted for 99% (bottom sediment) and 83% (pore water) of the total variance in the set of the analyzed results. The positive loadings were observed among metals and PAHs, which suggested similar sources of anthropogenic origins. Moreover, its tributary—the Rivers Wisłok—is highly polluted with nutrients; the drainage basin here is mainly agricultural, with a few industrial centers.

## Conclusions

We have presented the environmental risk associated with heavy metal and PAHs concentration in the bottom sediment on the basis of their ecotoxicological properties. We found the highest toxicity to organisms and the highest concentration of metals (Zn, Pb, Cd) and all PAHs in sediments at station S1 (inlet, backwater station) and the lowest at station S2 (middle). Sediments from the Rzeszów Reservoir are moderately contaminated with heavy metals and PAHs, which is a result of industry (combustion processes) and the impact of transportation in the area of the reservoir location. Clay content and organic C content are likely to be important factors, which control heavy metal and PAH concentrations in sediments. Moreover, positive correlations between individual pairs of heavy metals and PAHs prove that they have a similar source and move together to the reservoir sediments. The PEC quotients of the six metals and eight PAHs indicated a high potential toxicity of sediments from stations S1 and S3 and a low potential toxicity of sediments from station S2. We found that the bottom sediments from the Rzeszów reservoir were toxic. All bottom sediments and solid phases were classified as toxicity class III (acute toxicity) and II (slight acute toxicity). The pore water samples were classified as toxicity class I (no acute toxicity) and II (slight acute toxicity). The tested organisms presented different sensitivities. The most sensitive organism for metals and PAHs in bottom sediments was *L. sativum,* and in pore water—*S. saccharatum*. The phytotoxicity of the sediments for *S. alba* and *S. saccharatum* was caused mainly by Cd. The concentration of metals and PAHs in bottom sediments did not affect the toxicity for *H. incongruens* and *V. fischeri*.

In summary, the innovative potential of our results—a battery of biotests with organisms from different trophic levels—allows for a better sediment toxicity assessment. The integration of ecotoxicological and chemicals methods is necessary for an appropriate ecological risk assessment, and it presents satisfactory results. The results of this study provide a useful aid for sustainable reservoir management in the region.
